# A catalytic ozonation process using MgO/persulfate for degradation of cyanide in industrial wastewater: mechanistic interpretation, kinetics and by-products

**DOI:** 10.1039/d1ra07789a

**Published:** 2021-11-19

**Authors:** Ali Behnami, Jean-Philippe Croué, Ehsan Aghayani, Mojtaba Pourakbar

**Affiliations:** Department of Environmental Health Engineering, Maragheh University of Medical Sciences Maragheh Iran ppourakbar@yahoo.com +98 4132726363; Institut de Chimie des Milieux et des Matériaux, IC2MP UMR 7285 CNRS, Université de Poitiers France; Research Center for Environmental Contaminant, Abadan University of Medical Sciences Abadan Iran

## Abstract

Cyanide-laden wastewaters generated from mining and electroplating industries are extremely toxic and it is of vital importance to treat them prior to discharge to receiving water resources. The present study aims to oxidize cyanide using an ozonation process catalyzed by MgO and persulfate (PS). A MgO nanocatalyst was synthesized using the sol–gel method and characterized. The results show that the synthesized catalyst had a BET surface area of 198.3 m^2^ g^−1^ with a nanocrystalline particle size of 7.42 nm. In the present study, the effects of different operational parameters were investigated, and it was found that the MgO/O_3_/PS process is able to oxidize 100 mg L^−1^ of cyanide after 30 min under optimum operational conditions. Cyanide degradation mechanisms in the MgO/O_3_/PS process were completely investigated and the main radical species were identified using scavenging experiments. It was found that sulfate and hydroxyl radicals both contributed to the cyanide degradation in the MgO/O_3_/PS process. Cyanide degradation by-products were also tracked and it was found that cyanate and ammonium species are primarily generated during the oxidation, but increase of reaction time allowed their conversion to much less toxic compounds such as nitrate and bicarbonate. Cyanide degradation was also conducted in real industrial wastewater containing 173 mg L^−1^ of cyanide. Although there was a reduction in cyanide removal rate, the MgO/O_3_/PS process was able to completely oxidize cyanide within 70 min. Finally, it can be concluded that the ozonation process catalyzed by MgO and persulfate is an efficient and reliable advanced oxidation process for removal of cyanide from industrial wastewater.

## Abstract 2

Cyanide-laden wastewaters are extremely toxic and it is of vital importance to treat them prior to discharge to receiving water resources.

## Graphical abstract

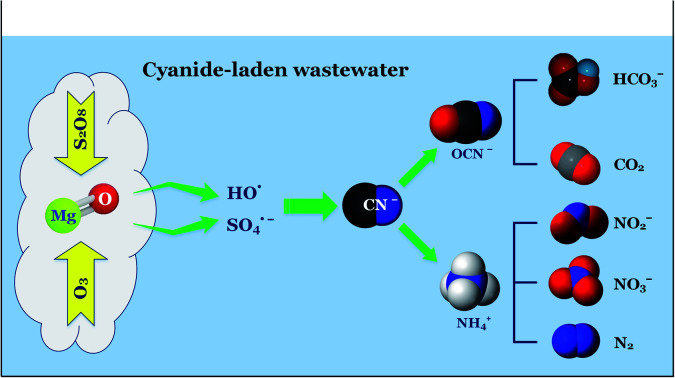

## 1. Introduction

Cyanide is a toxic compound that has been widely found in wastewater generated from various industries such as pharmaceuticals, mining, metal plating, gold extraction and dye production. Due to extremely dangerous effects to humans and other living organisms, cyanide is classified as a priority pollutant by the U.S. Environmental Protection Agency.^1,2^ The maximum permissible cyanide concentration in wastewater effluent is recommended to be below 0.2 mg L^−1^ prior to discharge into natural water bodies.^3^ Hence, to comply with strict environmental legislations, industries are required to provide adequate treatment methods for removing cyanide from wastewater.

In order to remove cyanide from the cyanide-laden wastewaters, several conventional physical, chemical, biological and adsorption processes have been investigated. Although the biological processes are efficient, cost-effective and widely used methods for industrial and municipal wastewater treatment,^4^ the toxicity of cyanide is a potent inhibitor for microbial growth and activity,^5^ which results in low biodegradation rate of cyanide. Physical treatment technologies such as membrane separation and ion exchange are very expensive, and they only transfer cyanide from one phase to another. Cyanide adsorption onto pistachio hull wastes,^4^ synthetic zeolite,^6^ and coke breeze^7^ were investigated previously and showed acceptable removal efficiency. However, adsorption processes can be expensive due to adsorbent, regeneration and waste management costs. Chemical methods such as alkaline chlorination are widely used for treatment of cyanide-containing wastewater, but they are often expensive, generate sludge and hazardous by-products.^8,9^

In recent years, many studies have been focusing on removing cyanide and its related degradation by-products from industrial wastewaters using Advanced Oxidation Processes (AOPs). Considering the toxicity of cyanide and its derivatives to microbial communities, AOPs can be suitable processes for treating these bio-refractory pollutants. AOPs utilize O_3_, persulfate (PS) and H_2_O_2_ enhanced with catalysts, UV light and electrochemical methods to generate highly potent hydroxyl radicals.^10^ Hydroxyl and sulfate radicals (HO˙ and SO_4_˙^−^) have almost similar redox potential (HO˙: *E*^0^ = 2.74 V and SO_4_˙^−^: *E*^0^ = 2.5–3.1 V) leading fragmentation of pollutants and mineralization to CO_2_ and H_2_O. However, in comparison to HO˙, SO_4_˙^−^ has some advantages including broad operational pH range (2–8) and longer half-life (30–40 μs) which provides better contact chance with contaminants. SO_4_˙^−^ can be generated through the activation of PS *via* various methods including heat, catalyst, electrochemical, UV, *etc.* SO_4_˙^−^ can efficiently degrade target pollutants present in aqueous solutions.^11,12^

So far, various AOPs are employed to remove cyanide from wastewater including ozone oxidation,^13,14^ photocatalytic processes,^9,13,15^ Fenton oxidation,^16,17^ and catalytic ozonation^18^*etc.* Among them, Catalytic Ozonation Process (COP) has been considered as an attractive and promising ozone-based AOP. In COP, the presence of metal oxides such as Fe, Mn, Mg, Ni, Ce and Co accelerate the decomposition of ozone and enhance the production of hydroxyl radicals, thereby increasing the degradation rate of refractory and also ozone-recalcitrant compounds.^19^ A variety of metal base catalysts such as ZnO,^20^ (MnO_2_–Co_3_O_4_)/AC,^21^ Fe_2_O_3_,^22^ MgO,^23^*etc.*, have been investigated for their efficiency to activate O_3_ for refractory pollutants degradation.

Among the aforementioned materials, MgO has received significant attention in conventional wastewater treatment due to its non-toxic and adsorption properties, because it is environmentally friendly, economical, and showed high stability in water.^24,25^ Based on literature review, to date, MgO has been reported as a capable catalyst to efficiently catalyze O_3_ to degrade a variety of compounds including antibiotics and drugs,^23,26^ organic compounds^27,28^ and dye^29,30^ in water environment.

To our knowledge, no study has been conducted to investigate the oxidation of cyanide in the ozonation process catalyzed by MgO in the presence of persulfate for simultaneous generation of sulfate and hydroxyl radicals. Therefore, this study aims to analyze the effect of the main operational variables, including catalyst concentration, cyanide concentration, and reaction time, on the removal of free cyanide synthetic wastewater and then real chromium electroplating wastewater. Degradation by-products are thoroughly tracked in the process and various mechanisms are proved in the MgO/O_3_/PS for free cyanide removal.

## 2. Materials and methods

### 2.1. Reagents and materials

Cyanide solutions were prepared from NaCN salt. The pH of the all solutions containing NaCN salt was adjusted to 11 by NaOH solution. The real wastewater used in this study was taken from a chromium electroplating plant. Chemicals used in the experiments were of the analytical grade and purchased from Merck Co. All chemicals were used without further purification.

### 2.2. Preparation of catalyst

In this study, MgO was prepared by sol–gel method. Magnesium acetate tetra-hydrate (Mg (C_2_H_3_O_2_)_2_·4H_2_O) was used as magnesium source. First, 50 g of Mg(C_2_H_3_O_2_)_2_·4H_2_O were dissolved in 500 mL of distilled water and gently stirred for 30 min. NaOH 1 N was used as alkaline agent to increase pH and form the gel. The solution was then stirred for 4 hours to form magnesium hydroxide (Mg(OH)_2_) precipitates. In order to separate Mg(OH)_2_ deposits, the suspension was stirred at 3000 rpm for 5 min. The separated deposits were washed three times using a mixture of ethanol and distilled water (50/50 vol% ratio) for removal of residual impurities from the prepared gel, and dried at 60 °C for 24 hours. Finally, the dried powder was collected and calcinated in an oven at 500 °C for 2 hours to form MgO nanoparticles.^23^

### 2.3. Characterization techniques

The characteristics of the synthesized catalyst such as the surface morphology of the catalyst were investigated using Scanning Electron Microscopy (SEM) coupled with energy dispersive X-ray spectroscopy (EDX). The specific surface area and the pore size of the catalyst were determined by the Brunauer–Emmett–Teller (BET) technique by the N_2_ adsorption/desorption method. X-ray diffraction (XRD) technique was used to analyze the crystalline phase of the MgO using Cu Kα radiation, scanned for 2*θ* of 10°–90°. The structural composition of the catalyst was examined by X-ray fluorescence (XRF). Fourier Transmission Infrared Spectroscopy (FTIR) was also used to determine the functional groups on the surface of the catalyst. Point of zero charge pH (pH_pzc_) was also measured for the synthesized MgO using the pH-drift method. In this characterization experiment, 100 mL of 0.1 M NaCl solution was used at ambient temperature. The pH of the solution was adjusted between 2–13 using NaOH or HCl solutions. Then, 100 mg of the MgO was added and stirred for 24 h. The final pH (pH_final_) of the solution was plotted in front of initial values of pH (pH_initial_). Finally the point at which final and initial pH were the same was selected as pH_zpc_.^31^

### 2.4. Experimental procedure

**Fig. 1:**
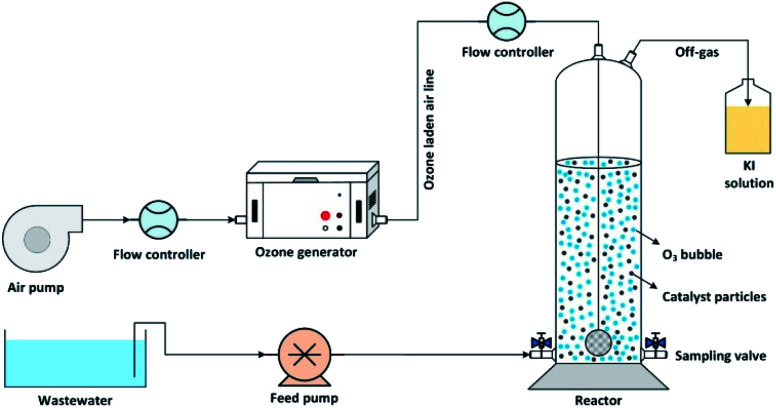
Schematic diagram of the experimental setup.

The catalytic ozonation of wastewater using MgO as a catalyst was performed in batch mode in a lab-scale cylindrical reactor as shown in Fig. 1. Ozone gas was produced using an ozone generator (ARDA model) and uniformly bubbled into the solution using glass diffuser at the bottom of the reactor. The inlet ozone concentration was controlled by adjusting the airflow rate passing through the ozone generator. The residual ozone in the off-gas stream was trapped into a potassium iodide (KI) solution.

This study can be divided into two main parts: firstly, the effect of the MgO/O_3_ process in cyanide removal, secondly, investigating the benefit of adding PS to the MgO/O_3_ process (MgO/O_3_/PS process). To evaluate the performance of aforementioned processes, adsorption rate of cyanide onto the MgO catalyst, the effect of the Sole Ozonation Process (SOP), Catalytic Ozonation Process (COP), and persulfate (PS) alone were also studied. Accordingly, the effect of operational variables such as ozone dosage, MgO concentration, cyanide and anions (including carbonate, bicarbonate, nitrate, sulfate, phosphate and chloride) concentrations, and the effect of contact time in MgO/O_3_ and MgO/O_3_/PS processes were examined to determine the optimum operational conditions. To determine radical based degradation of cyanide, the effect of radical quenchers such as *tert*-butanol (TBA), and ethanol (EtOH) was examined. Radical scavengers were used at the concentration of 5 g L^−1^. A comprehensive investigation was also carried out with different combination of oxidizing agents to identify the possible degradation mechanisms of the degradation of cyanide. All the experiment were conducted in a solution pH of 11, since the dissolved cyanide is converted into gaseous HCN and released in the lower pH values.

To predict the cyanide degradation mechanism, the influence of possible by-products such as cyanate (OCN^−^), ammonium (NH_4_^+^), nitrite (NO_2_^−^), nitrate (NO_3_^−^), and alkalinity were measured under the optimum experimental conditions. In addition, residual persulfate concentration and sulfate in the effluent were measured.

Finally, in order to evaluate the practicality of the processes in actual application, the performance of MgO/O_3_/PS process for cyanide removal was investigated in different water matrices in the presence of water anions (nitrate, chloride, sulfate, phosphate, carbonate), tap water, and the real chromium electroplating wastewater. Table 1 shows the composition of studied wastewater. To ensure the accuracy and reliability of the results, two replicates of each experiment were conducted and the average values of the measurements have been presented.

**Table 1:** Characteristics of wastewater

Parameter	COD (mg L^−1^)	pH	CN (mg L^−1^)	Cr (mg L^−1^)	TSS (mg L^−1^)
Value	976	10.8	173	63	101

### 2.5. Analytical methods

Due to the interference of cyanide with impurities in the samples such as persulfate and yellowish colour of the chromium electroplating wastewater, cyanide was extracted from the solution in accordance with the standard methods for the examination of water and wastewater. In this method, acetic acid was added to the solution and the pH was reduced to 3. This resulted in formation of gaseous HCN. The generated HCN was then diffused into the NaOH normal solution. Finally, the cyanide content in the solution was measured by titrimetric method using a standard solution of silver nitrate in the presence of *p*-dimethylaminobenzalrhodanine indicator.^32^

In order to determine the fate of cyanide in the MgO/O_3_/PS process, the concentration of cyanate, nitrite, nitrate, ammonium, bicarbonate, and sulfate were analyzed following the Standard Methods.^32^ Iodometric method was used to quantify the residual persulfate in the effluent.^33^

The ozone concentration in the inlet and the process off-gas was measured by sparging the gas stream into a potassium iodide solution followed by iodometric titration.^32^

## 3. Results and discussion

### 3.1. Catalyst characterization

**Fig. 2:**
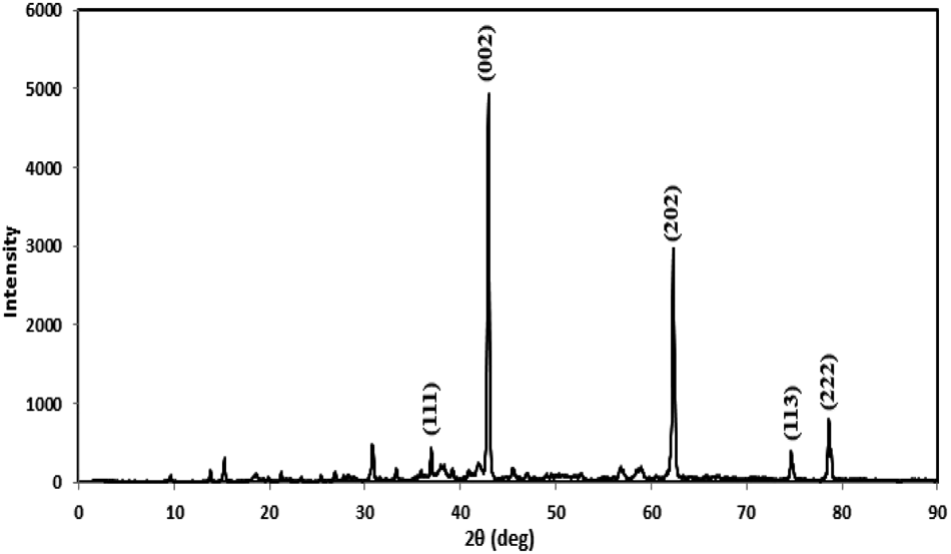
The XRD pattern of the synthesized MgO.

Prior to conducting the oxidation experiments, the synthesized MgO was fully characterized. Fig. 2 is illustrating the XRD pattern of the prepared MgO. As it is shown in the figure there are sharp distinguished peaks at 2*θ* of 36.97°, 43.03°, 62.29°, 74.67°, and 78.61° corresponding to the (111), (002), (202), (113), and (222) planes, respectively (JSPDS no. 87-0653). The XRD patterns of the MgO showed a well crystallized material owing to the high intensity, narrow width and sharpness of diffraction peaks. The mean crystallite size of the catalyst was also calculated using the Debye–Scherrer's formula.^34^1
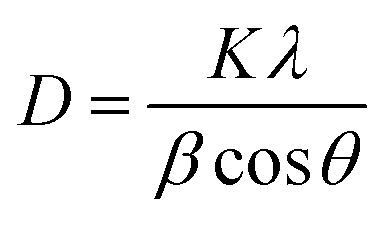
where *D* is the mean crystallite size, *λ* is the wavelength of Cu Kα radiation, *β* is the Full Width at Half Maximum (FWHM), *θ* is the angle of reflection, and *K* is the shape factor (0.9). Based on eqn (1), *D* was calculated to be 24.5 nm for the highest peak at (002) plane. The synthesized MgO showed similar XRD pattern as the one published in other studies.^35,36^ Balakrishnan *et al.*^35^ synthesized MgO by the solution combustion method. The XRD pattern was also similar to our study and they found the highest peak at (002) plane, and the mean crystallite size was also reported to be 27 nm which is close to the value obtained in our study. Ismail Ercan *et al.*^36^ synthesized MgO nanoparticle using the combustion method, the XRD results also showed a similar pattern with a mean crystallite size of 26–37 nm.

**Fig. 3:**
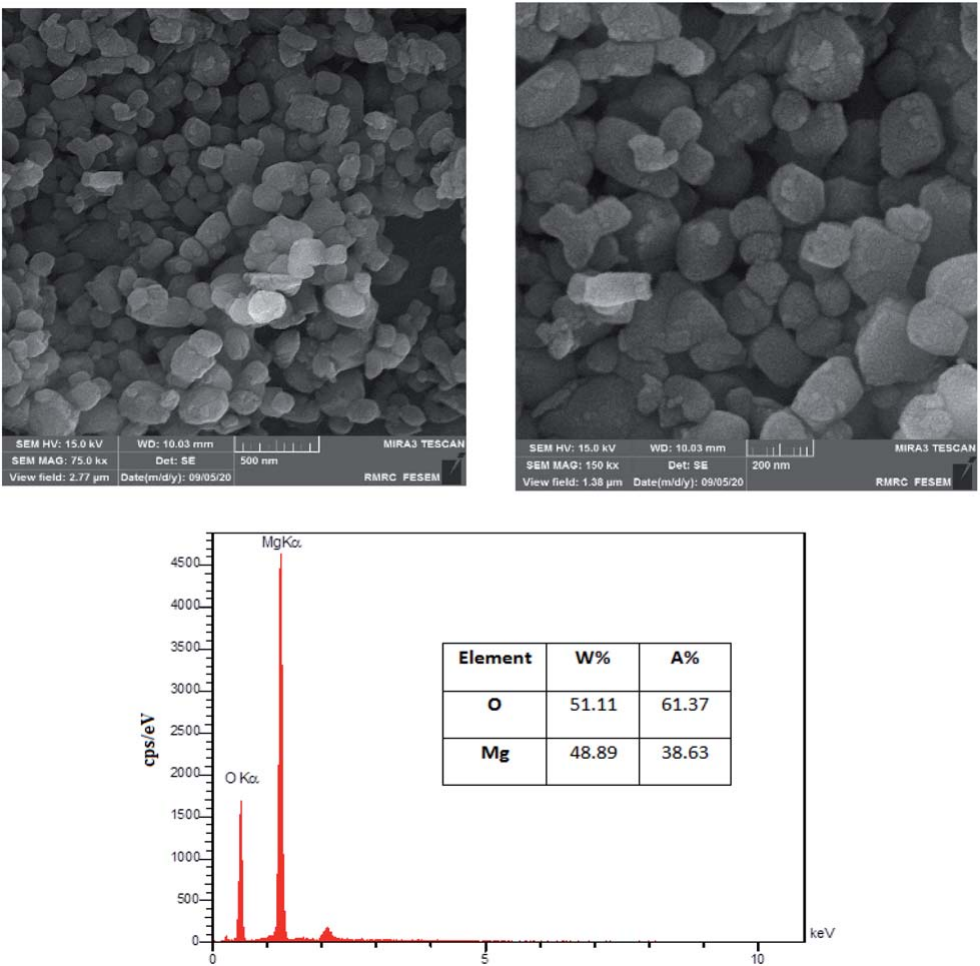
FESEM images at different magnifications, and EDX spectra of synthesized catalyst.

The surface morphology of the MgO was investigated using FESEM images. Fig. 3 is illustrating the MgO catalyst structure at different magnifications (75k× and 150k×). The formation of nanoparticles are clearly observed in the high resolution SEM images. The nanoparticles are in spherical shapes, uniformly distributed and highly porous with agglomeration.^37^ In order to investigate the elemental characterization of the MgO particles, the EDX technique was used. The results of EDS analysis are also illustrated in Fig. 3. As it is shown in the figure, there is no impurity in the synthesized MgO. EDS results showed that the weight percentages of magnesium and oxygen were 48.89% and 51.11%, respectively.

XRF technique was used for the identification of the chemical composition of the MgO catalyst (Table 2). Based on the results, MgO was the main compound (70.8%) found in the catalyst which is in accordance with the results of XRD and EDS. Based on the experimental data, almost 28% of the catalyst has burned and missed in the XRF technique which is reported as LOI (Loss of Ignition).

**Table 2:** Chemical composition of the synthesized MgO

Composition	MgO	Cl	CaO	Fe_2_O_3_	LOI^a^	Total
Quantity (%)	70.8	0.2	0.13	0.06	28.81	100

a Loss of ignition: the catalyst was heated at 950 °C for 90 min.

**Fig. 4:**
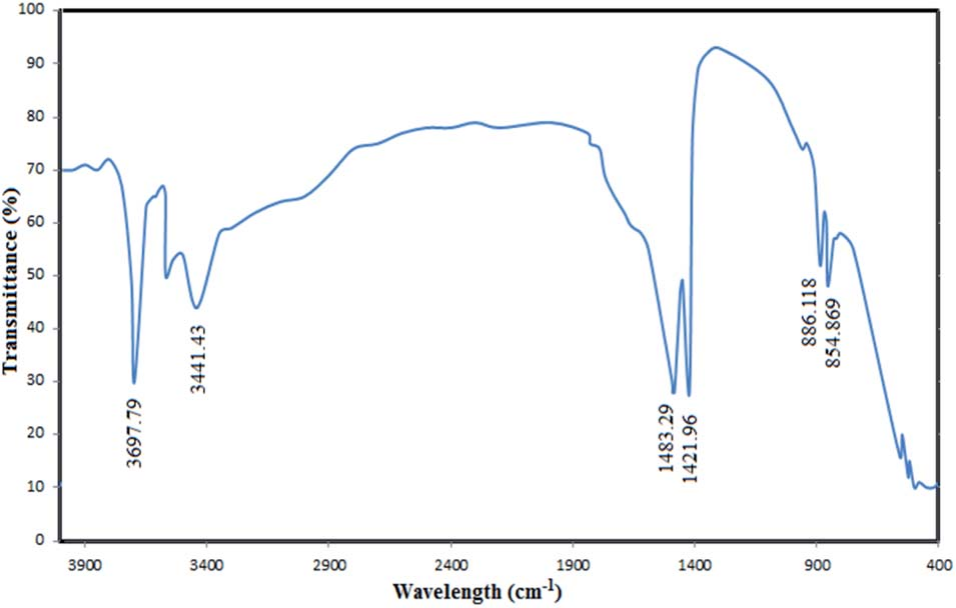
The FTIR spectra of the synthesized MgO.

To confirm the successful synthesis and analyse the chemical structure of the MgO nanoparticles, FTIR spectrum of the catalyst was recorded (Fig. 4). The absorbance values were carefully investigated in the FTIR spectra to understand the surface properties of the synthesized MgO. As shown in the figure, the bands observed at 854 cm^−1^ and 886 cm^−1^ correspond to the vibration of Mg–O bands which have also been reported in similar MgO spectra in the literature.^38^ The distinct bands observed at 1421 cm^−1^ and 1483 cm^−1^ are associated to the vibration of surface hydroxyl groups.^35^ The observed broad bands at 3441 cm^−1^ and 3697 cm^−1^ are due to the OH stretching vibration of water molecules.^39^

It is of great importance to evaluate the point of zero charge pH of the catalyst. The pH-drift procedure was selected to find pH_pzc_. The pH_pzc_ was measured to be 12.4 for the synthesized MgO (data not shown). This value is close to the values reported in literature. *E.g.* Chen *et al.*^28^ measured the pH_pzc_ of various MgO nanoparticles synthesized with different methods. The pH_pzc_ values were all reported to be 12.1–12.7. Mashayekh-Salehi *et al.*^23^ also reported pH_pzc_ of 10.9 for the synthesized MgO.

**Fig. 5:**
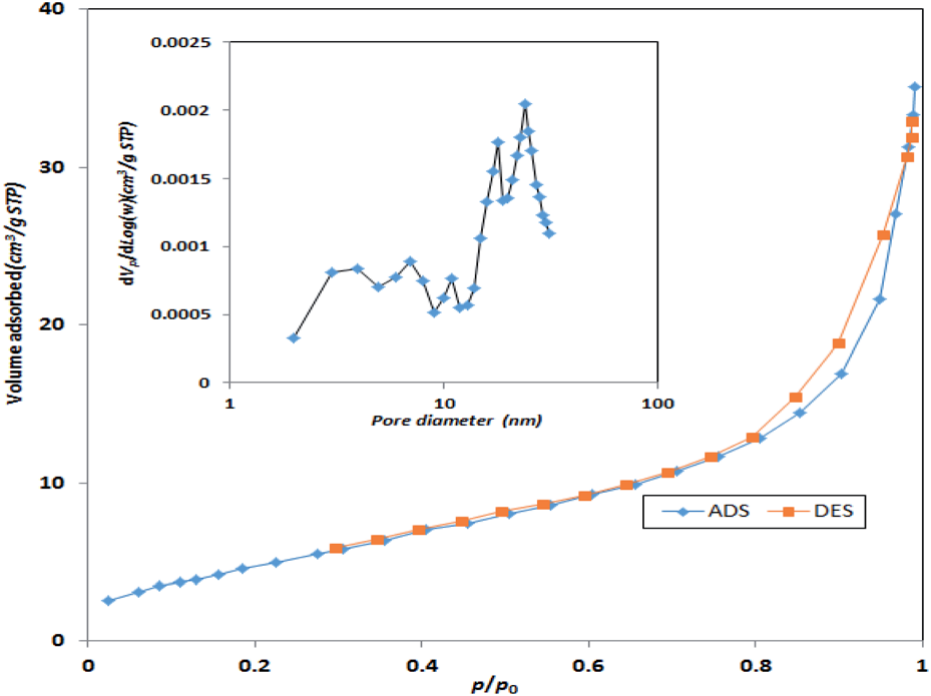
N_2_ adsorption–desorption isotherms of MgO catalyst.

Specific surface area and average pore size of the synthesized catalyst were also measured using BET surface area. Based on Fig. 5, the specific surface area of the synthesized MgO was 198.3 m^2^ g^−1^ with an average pore size of 7.42 nm. Therefore, the synthesized MgO could be considered as a mesoporous material (IUPAC classification). The higher surface area of the catalyst could be a determining factor for the enhancement of catalytic activity. The specific surface area and average pore size of the current MgO is close to the values reported in other studies.^23^

### 3.2. Contribution of different oxidation processes for cyanide removal

**Fig. 6:**
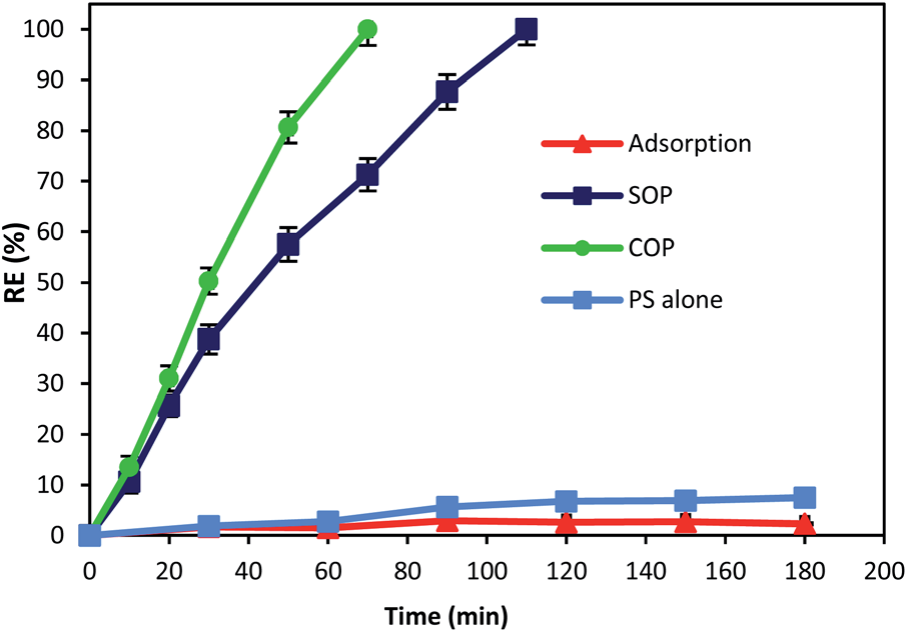
Contribution of adsorption, SOP, COP and PS alone on cyanide removal (cyanide concentration: 100 mg L^−1^, MgO concentration: 500 mg L^−1^, ozone concentration: 200 mg h^−1^, PS concentration: 200 mg L^−1^, pH = 11, reaction time: 180 min).

The catalytic activity of MgO was tested for the oxidation of cyanide. In this regard, preliminary experiments were carried out to describe the contribution of different processes (adsorption, SOP, COP and PS) in cyanide removal using reaction time as an operational variable. Fig. 6 displays the contribution of different processes in cyanide removal. As can be seen in the figure, adsorption has low contribution in cyanide removal (less than 3%), indicating that the effect of adsorption on the COP can be neglected. In previous studies, MgO has been reported as a cost-effective and potential adsorbent for the removal of drugs,^40^ heavy metals,^41,42^ and various anions.^43,44^ However, Fig. 6 revealed that the MgO catalyst is not an effective adsorbent for cyanide. Based on the results, the adsorption rate of cyanide first slightly increased (2.9%, at min 90), then decreased to 2.3% (at min 180). There is no similar study using the MgO catalyst for cyanide adsorption. However, there are some other studies using MgO for other contaminants adsorption. For instance, Chen *et al.*^28^ reported that only less than 3% of 4-chlorophenol was adsorbed onto four different MgO catalysts that they investigated. Mashayekh-Salehi *et al.*^23^ also revealed that adsorption rate of acetaminophen onto MgO nanoparticles was around 1% at the contact time of 15 min. The reduction in adsorption rate can be attributed to the limited available space for cyanide adsorption on MgO and therefore, the exterior surface of MgO particles are saturated with cyanide molecules in the solution. However, adsorption of cyanide onto MgO is not major factor for effective COP performance.

As shown in Fig. 6, after 180 min of reaction, the cyanide removal rate reached 7.5% for PS alone. Although PS has high redox potential of 2.01 V,^45^ PS alone had no significant effect on cyanide removal at pH 11. The possible reason can be the absence of PS activating agent which causes PS to remain nearly intact in the solution and does not actively participate in cyanide removal. This is in accordance with similar studies using PS alone for cyanide containing compounds degradation. Castilla-Acevedo *et al.*^46^ exposed 1 g L^−1^ of PS at pH 11 to cyanide containing wastewater and only a negligible degradation has occurred. Therefore, it can be concluded that activation of PS in alkaline solution is not contributing for PS activation and cyanide degradation.

Based on the results, it can be seen that ozonation alone could degrade cyanide completely after 110 min of reaction. Presence of ozone in the cyanide-laden solution could lead to the degradation of cyanide through two main reaction pathways. Firstly, ozone could directly react with cyanide molecules and produce cyanate, nitrogenous compounds and bicarbonate as given in eqn (2) and (3):^14^

2 3CN^−^ + O_3_(aq) → 3OCN^−^

3 2OCN^−^ + O_3_(aq) + H_2_O → 2HCO_3_^−^ + N_2_

Secondly, under alkaline conditions (alkaline ozonation) ozone can mainly decompose to HO˙, which react with the target compound. It has also been reported that under basic condition, HO˙ is the main oxidizing agent in the solution.^47^ Therefore, second reaction pathway through generated radicals is the main degradation process in SOP. Although SOP can completely destruct cyanide (Fig. 6), it needs longer reaction time and consequently higher energy consumption, since more electric power is required at higher reaction times.

### 3.3. Catalytic activity of MgO in COP

As seen in Fig. 6, combination of catalyst and the ozonation process significantly improved the cyanide degradation rate. COP reached 100% cyanide removal rate in the lower reaction time (70 min). This is due to the synergetic effect of ozone and MgO, and formation of highly active radicals. To understand the synergistic effect of catalyst addition to the ozonation process for effective degradation of cyanide, the following equation was used:

4 Synergy (%) = % removed in COP − [% removed in SOP + % removed by adsorption]

According to the results, the highest synergy of 26.74% was calculated for MgO catalyst on the degradation of cyanide in combination with the ozonation process at a reaction time of 70 min. The synergistic effect of a combined method of ozonation and MgO catalyst increased from 3.88% to 26.74% with the increase of reaction time from 20 min to 70 min. Considering that the highest adsorption rate of cyanide onto MgO nanoparticles was negligible (2.9%, at min 90), therefore it can be deduced that the cyanide was mainly removed through degradation. The findings indicate the high potential of MgO catalyst to catalyze the ozonation process, thereby improving the cyanide degradation rate in COP.

In COP, besides the direct reaction of cyanide with ozone (eqn (2) and (3)) molecules, the enhanced removal rate of cyanide can be described to the adsorption of ozone molecules on the surface of MgO nanoparticles and subsequently decomposition into active radicals.^29^ As mentioned before, ozone molecules are unstable at pH 11, and also they can initiate reactions directly with cyanide molecules. However, since there are higher concentrations of injected ozone in the inflow gas stream, the MgO activation is also favored in the COP. The following equation describe the direct oxidation of cyanide with ozone on MgO surface:

5 MgO^–O_3_^ + CN → CO_2_ + H_2_O + intermediates

The presence of basic functional groups (hydroxyl groups or isolated oxygen) on the surface of nanosized MgO play an essential role in transformation of ozone molecules to active hydroxyl radicals.

On the other hand, pH_pzc_ of the MgO was found to be 12.4, and pH of the cyanide-laden solution are below 11. Therefore, the catalyst surface will be positively charged (Mg–OH_2_^+^) in the cyanide solution. The positive surface of the catalyst will attract the OH^−^ and will form a basic microenvironment around the MgO catalyst in the cyanide-laden solution. Presence of high amounts of OH^−^ around the catalyst will eventually lead to the formation of more HO˙ due to the ozonation of the solution, since OH^−^ acts as an initiator of radical formation.^28^

### 3.4. Persulfate activation in MgO/O_3_ process

**Fig. 7:**
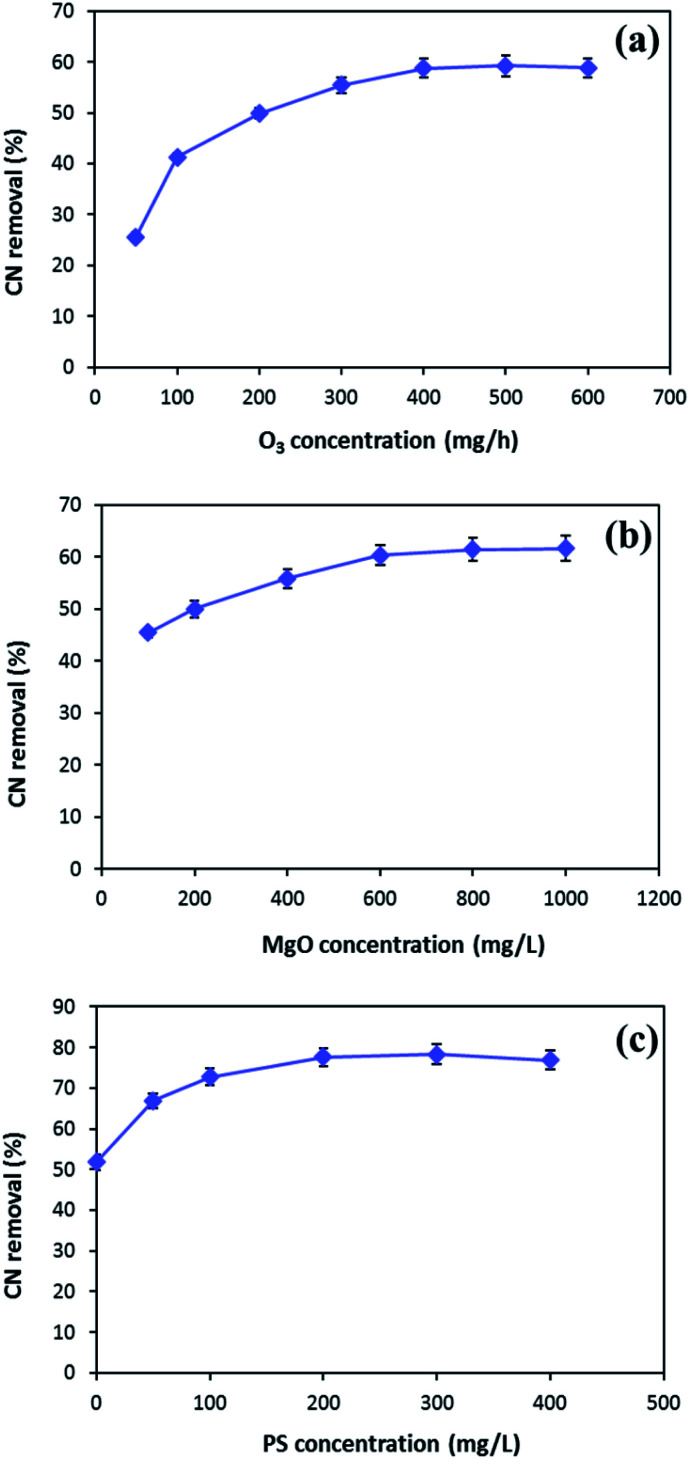
The effects of ozone concentration (a) and MgO dosage (b) in MgO/O_3_ process and PS concentration (c) in MgO/O_3_/PS process (MgO = 500 mg L^−1^ in (a), ozone = 400 mg h^−1^ in (b), time = 30 min) (MgO = 600 mg L^−1^, ozone = 400 mg L^−1^, time = 20 min in (c)).

In order to enhance the MgO/O_3_ process, precursor of sulfate radical is added to the COP, and a thorough investigation of the effective parameters on the process performance was conducted. Optimization of the developed processes was conducted using synthetic wastewater to determine the optimum operating condition for effective cyanide removal (100 mg L^−1^). To find out the optimum injected ozone dosage, different concentrations of ozone were used in the MgO/O_3_ process. The effect of ozone dosage on the removal of cyanide is shown in Fig. 7a. As can be seen in the figure, the cyanide concentration steeply decreased by increasing injected ozone concentration up to 400 mg h^−1^, beyond which no significant change in degradation rate was observed. Therefore, the optimum injected ozone dosage was found around 400 mg h^−1^. This might be ascribed to the fact that further increase in the injected ozone concentration can result in the reaction of ozone with HO˙ and causing the self-scavenge effect. Furthermore, higher concentrations of ozone could not completely dissolve in the solution and the excess ozone will be released in the off-gas form.^48,49^

The catalytic activity of MgO in the solution was evaluated for cyanide oxidation in the MgO/O_3_ process. To optimize the concentration of catalyst, various amounts of MgO ranging from 100–1000 mg L^−1^ were used. Fig. 7b depicts the effect of the MgO catalyst on cyanide removal in the MgO/O_3_ process under condition of 400 mg h^−1^ injected ozone concentration and 30 min of reaction time. Based on the figure, the optimum MgO dosage in the MgO/O_3_ process for efficient removal of cyanide under our experimental conditions was obtained around 600 mg L^−1^. The increased degradation of cyanide in the MgO/O_3_ process can be attributed to the larger surface areas, more active sites, and formation of highly oxidizing radicals.^29,50^ However, further increasing in the MgO concentration (up to 600 mg L^−1^) did not considerably increase the cyanide removal rate. Beside the self-decomposition of hydroxyl radicals, this could be explained by the agglomeration of catalyst particles which reduces the number of active sites for production of hydroxyl radicals.^29^

In the second phase of study, persulfate, as a sulfate radical precursor, was added to the reaction. Optimization of PS concentration is an important factor since PS is the source of SO_4_˙^−^ in the solution. According to Fig. 7c, the highest cyanide removal efficiency (77.7%) was achieved when PS concentration was adjusted around 200 mg L^−1^. By increasing PS concentration from 200 to 400 mg L^−1^, no significant increase in cyanide removal efficiency was observed. This can be due to the fact that increasing PS concentration causes scavenging effect between generated radicals.^15^ Hence, the optimum PS concentration for efficient cyanide removal was 200 mg L^−1^.

Taking into account all the aforementioned results, the optimal reaction conditions were determined as follows: injected ozone concentration of 400 mg h^−1^, MgO concentration of 600 mg L^−1^, and PS concentration of 200 mg L^−1^.

### 3.5. The effect of reaction time and cyanide concentration

**Fig. 8:**
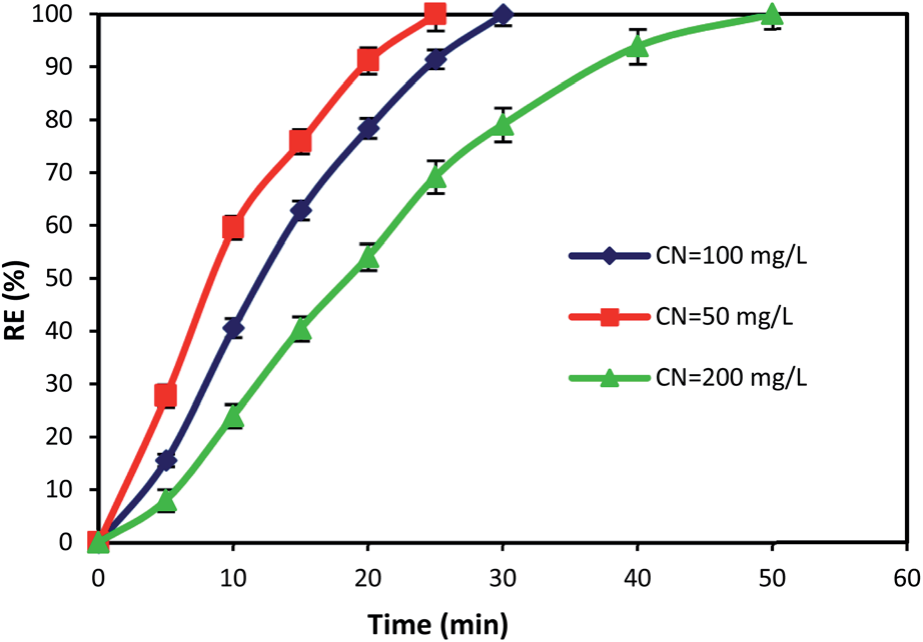
Effects of contact time and initial cyanide concentration on the removal of cyanide by MgO/O_3_/PS process.

The initial concentration of pollutants and reaction time are crucial factors that have a great influence on the design and operation performance of an oxidation process. Hence, in this study, the time dependent behavior of cyanide degradation by varying the reaction time in the range of 0–60 min with different initial cyanide concentration (50, 100, and 200 mg L^−1^) was investigated. Fig. 8 illustrates the cyanide removal *versus* the contact time under the optimum condition of MgO = 600 mg L^−1^, O_3_ = 400 mg h^−1^, and PS = 200 mg L^−1^ by the MgO/O_3_/PS process. Results showed that the cyanide removal rate in the MgO/O_3_/PS process decreased by increasing the initial cyanide concentration from 50 to 200 mg L^−1^. The complete degradation was achieved at 25 min contact time for a cyanide concentration of 50 mg L^−1^, but with increasing initial cyanide concentration from 50 to 200 mg L^−1^, it proceeded at a slower rate. Therefore, more contact time is required for degradation of high concentrations of input cyanide. The high removal rate in low cyanide concentration (50 mg L^−1^) is attributed to the presence of sufficient amount of reactive radicals in the solution. Moreover, increasing in initial cyanide concentration with constant initial concentrations of ozone, MgO and PS led to an increase of cyanide concentration to oxidizing agent's ratio, thereby reducing the removal efficiency.

### 3.6. Degradation kinetics and cyanide removal mechanism

In the sulfate radical based AOPs, SO_4_˙^−^ and HO˙ are simultaneously generated in the oxidation reaction. To identify the main mechanisms participated in the target pollutant degradation, it is important to find the principle reactive radical since it has direct implication of the process performance, degradation pathway, and degradation by-products. One of the commonly used methods for identification of the main oxidizing radicals is indirect competitive kinetic method using scavenging agents. Usually TBA and EtOH are used to identify the main oxidizing radicals in the sulfate based AOPs. It has been demonstrated that EtOH can react with both HO˙ (*K*_EtOH·HO˙_ = (1.2–2.8) × 10^9^ M^−1^ s^−1^) and SO_4_˙^−^ (*K*_EtOH·SO_4__ = 1.6 × 10^7^ M^−1^ s^−1^). On the other hand, TBA is a reliable HO˙ radical quencher.^51^ The reaction rate constant of TBA and HO˙ is (3.8–7.6) × 10^8^ M^−1^ s^−1^ which is 1000 times more than that of SO_4_˙^−^ (*K*_TBA·SO_4__ = (4–9.1) × 10^5^ M^−1^ s^−1^). Therefore, TBA and EtOH are used as radical capturing agents to identify the relative contribution of the SO_4_˙^−^ and HO˙.

In addition, to better elucidate the potential of catalyst for catalyzing the cyanide degradation in the ozonation process, the rate constant of the cyanide oxidation was determined following a pseudo-first order (PFO) kinetic model as follows:^9^6
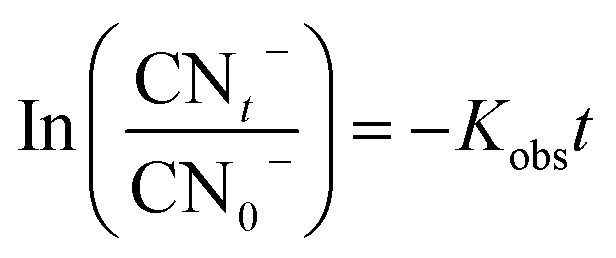
where CN_0_ represents the cyanide concentrations at the reaction beginning, CN_*t*_ is the cyanide concentration at time *t*, and *K*_obs_ is the PFO rate constant.

Cyanide degradation in the MgO/O_3_/PS process could be occurred through various degradation mechanisms. To better understand and prove the degradation pathways, various combinations of oxidizing agents were investigated for cyanide degradation. Following are the investigated degradation mechanisms:

- Adsorption on MgO catalyst.

- Oxidation by persulfate alone.

- Single Ozonation Process (SOP).

- MgO/O_3_.

- PS/O_3_.

- MgO/PS.

- MgO/O_3_/PS.

**Fig. 9:**
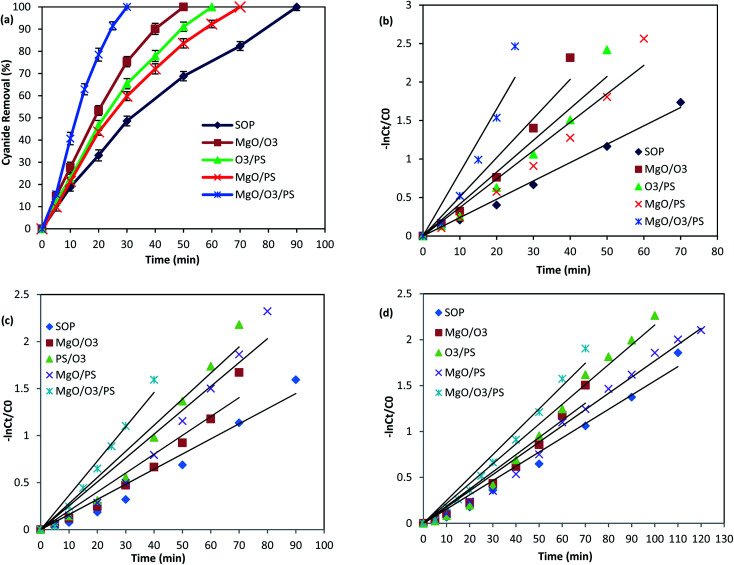
Cyanide removal efficiency by different AOPs without any scavenger (a), PFO kinetics without any scavenger (b) PFO kinetics in the presence of TBA (c) PFO kinetics in the presence of EtOH (d).

As it was illustrated in Fig. 6, the performance of adsorption and persulfate alone are negligible in cyanide removal efficiency, and therefore, they are not further investigated in the oxidation mechanism. However, the other remaining proposed mechanisms are thoroughly investigated to find the main oxidizing agents in the investigated processes. Fig. 9 is illustrating the performance of the above-mentioned pathways.

#### 3.6.1. Single ozonation process

As it is shown in Fig. 9a, SOP is able to oxidize 100 mg L^−1^ of cyanide after 90 min of reaction time. Since the cyanide-laden wastewater are all the time in the alkaline condition, therefore, generation of hydroxyl radical and direct oxidation of cyanide by ozone molecules could be the reasons of cyanide degradation. To prove the radical based degradation of cyanide, the reaction was conducted in the presence of excessive amount of scavengers. Fig. 9c is illustrating the PFO kinetic model of reactions in the presence of TBA. As it is shown in the figure, there is a reduction in reaction rate constant in the presence of TBA and also EtOH (Table 3). Therefore, oxidation of cyanide takes place through hydroxyl radical in SOP. Kiril Mert *et al.*^14^ reported oxidation of 100 mg L^−1^ of cyanide using SOP with ozone concentration of 180 mg h^−1^. They also confirmed that about 86% of cyanide was degraded by generated radical species in the SOP.

**Table 3:** Reaction rate constants of different contributing degradation mechanism in cyanide oxidation

Process	Without scavenger		TBA as scavenger		EtOH as scavenger		Contribution of HO˙	Contribution of SO_4_˙^−^
	*K*_obs_	*R* ^2^	*K*_obs_	*R* ^2^	*K*_obs_	*R* ^2^		
SOP	0.024	0.99	0.016	0.96	0.015	0.97	—	—
MgO/O_3_	0.056	0.97	0.020	0.95	0.018	0.95	—	—
PS/O_3_	0.045	0.96	0.027	0.95	0.021	0.97	42.56	57.44
MgO/PS	0.040	0.97	0.025	0.95	0.017	0.98	37.5	62.5
MgO/PS/O_3_	0.096	0.94	0.036	0.96	0.024	0.97	62.5	37.5

#### 3.6.2. MgO/O_3_

In the next experiment, the combination of MgO and ozone was investigated for cyanide oxidation. In this process, there could also be two different pathways for oxidation: (1) ozone direct oxidation (2) indirect oxidation by radical species generated through interaction of ozone molecules with catalyst surface. These two proposed mechanisms could occur simultaneously in the bulk solution or on the catalyst surface.^52^ To prove these mechanisms, cyanide degradation was carried out in the presence of radical scavengers. As it is summarized in Table 3, there is outstanding reduction in the *K*_obs_ values in the presence of TBA and EtOH, confirming the generation of hydroxyl radical in the investigated COP. The generation of HO˙ on the surface of catalyst could be due to the interaction of ozone molecules with the basic groups on the surface of synthesized MgO. This happens due to the electrophilic characteristics of the ozone molecules, and the basic functional groups of the catalyst such as OH^−^ which are finally transformed to the highly oxidizing HO˙.^53^ This mechanism are in consistence with the results of present study, as it has been demonstrated in Fig. 4, the OH functional groups are proved to be on the surface of synthesized catalyst due to the presence of strong broad bands at 3441 and 3697 cm^−1^. Considering the point that ozone benefits both nucleophilic and electrophilic sites, consequently it can interact with the both H (as electrophilic) and O (as nucleophilic) atoms of –OH groups on the surface of MgO nanoparticles. Therefore, it can be concluded that the hydroxyl groups are initiating the ozone decomposition on the catalyst surface leading to the accelerated generation of HO˙.^54^ In order to better illustrate the ozone decomposition on the catalyst surface, the ozone mass balance experiments were conducted. The injected ozone into the alkaline cyanide containing solution are consumed by the either hydroxide ions in the solution or functional hydroxyl groups of the MgO. Considering this point the consumed ozone was both measured in SOP and the MgO/O_3_ process. The results show that only 11.1% of ozone was consumed in SOP, while 20.5% of ozone was consumed in the MgO/O_3_ process, confirming that some part of the dissolved ozone was decomposed due to the interaction of ozone molecules with functional surface groups of the MgO nanoparticles.

#### 3.6.3. PS/O_3_

Persulfate activation by the ozone molecules is the other investigated mechanism in the present study. Activation of persulfate by ozone has already been used for oxidation of different organic contaminant.^55,56^ We also investigated the performance of this process for cyanide degradation (Fig. 9a). As it is shown in the figure, PS/O_3_ was also effective for cyanide removal and almost complete destruction of cyanide molecules occur within 60 min of reaction time. The ozone concentration in the outlet of this process was also investigated, and it was found that PS/O_3_ process is able to consume about 15.4% of the injected ozone into the process. The higher value of the consumed ozone compared with SOP (11.1%) is due to the consumption of ozone for PS activation.

In order to find the relative contribution of the SO_4_˙^−^ and HO˙, TBA and EtOH as radical quenchers were used. When excessive amount of TBA was added to the reaction, majority of the HO˙ were captured, and only small amount of SO_4_˙^−^ could react with cyanide molecules. In the second quenching experiment, excessive amount of EtOH was introduced into the process, based on the high reaction rate of EtOH with SO_4_˙^−^ and HO˙, both of them are simultaneously captured in the PS/O_3_ process. This is well-proved based on the reduction in the *K*_obs_ values in the presence of TBA and EtOH (Table 3).

The relative contribution of SO_4_˙^−^ and HO˙ in the sulfate based AOPs could be calculated by comparing the differences in the rate constants of pollutant removal with and without HO˙ scavenger. Accordingly, the contribution of SO_4_˙^−^ and HO˙ are calculated using eqn (7) and (8).^57,58^7
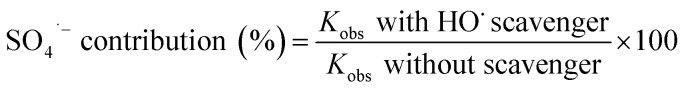

8 HO˙ contribution (%) = 100 − SO_4_˙− contribution (%)

Based on the reaction rate constants, it is found that almost 57.44% of the cyanide oxidation is due to the generation of SO_4_˙^−^ and 42.56% is due to the HO˙. Thus, it can be concluded that both SO_4_˙^−^ and HO˙ are the main oxidizing species in the PS/O_3_ process. Qiao *et al.*^55^ investigated the activation of persulfate by ozone molecules for degradation of cyanide. Based on the reaction rate constants it is found that almost 57.44% of the cyanide oxidation is due to the generation of SO_4_˙^−^ and 42.56% is due to the HO˙. Thus, it can be concluded that both SO_4_˙^−^ and HO˙ are the main oxidizing species in the PS/O_3_ process. Qiao *et al.*^55^ investigated the activation of persulfate by ozone molecules for degradation of nitrobenzene. They also used the same radical scavengers and proved the simultaneous generation of SO_4_˙^−^ and HO˙ in PS/O_3_ process.

#### 3.6.4. MgO/PS

**Fig. 10:**
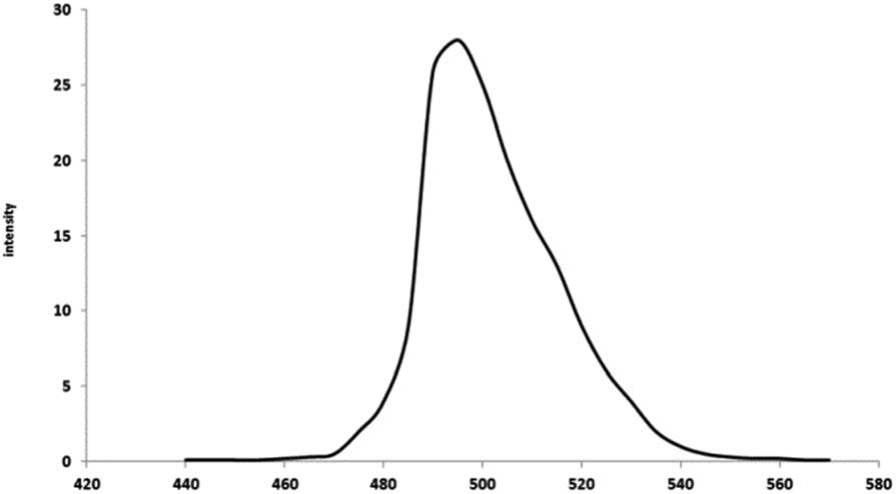
Photoluminescence of the synthesized MgO.

One of the other mechanisms of cyanide degradation in MgO/O_3_/PS process is the interaction between MgO catalyst and persulfate. Based on Fig. 5, persulfate and MgO alone were not effective for cyanide removal. However, as it is shown in Fig. 9a, complete removal of cyanide has been reached after 70 min of reaction time. Therefore, there should be an interaction between persulfate and MgO catalyst to generate much more oxidative compounds than persulfate. In order to find the mechanism of persulfate activation by MgO, photoluminescence properties of catalyst was investigated. Fig. 10 is illustrating the photoluminescence spectra of synthesized MgO. As it is presented in the figure, there is a broad sharp peak at wavelength of 495 nm. The observed peak is due to the oxygen vacancies on the surface of synthesized MgO catalyst.^35^ Presence of oxygen defects is favoring the valence band on the surface of catalyst and enhances the charge separation and transfer rate of electron and hole leading to the more oxidation of cyanide. The activation of persulfate by catalyst has also been reported in other studies. Guan *et al.*^59^ investigated persulfate and peroxymonosulfate activation by magnetic porous copper ferrite catalyst. They found that magnetic porous copper ferrite was not able to activate peroxymonosulfate, but persulfate activation has been reached which was due to the asymmetrical structure of the persulfate. Therefore, in our present study, asymmetric structure of the persulfate along with the surface oxygen vacancy of the MgO contribute to the formation of radical species. Subsequently, sulfate radical could transfer to the hydroxyl radical in alkaline solution as given in eqn (9).^15^

9 SO_4_˙^−^ + OH^−^ → SO_4_^2−^ + HO˙

Therefore, formation of both radicals are expected to occur in the solution. Table 3 is illustrating the scavenging tests results in MgO/PS process. Based on the *K*_obs_ values, the contribution of SO_4_˙^−^ is calculated to be 62.5% and contribution of hydroxyl radical is 37.5% in cyanide oxidation by MgO/PS process.

#### 3.6.5. MgO/O_3_/PS

The scavenging experiments were finally conducted on the most efficient process which has the most complicated mechanism for cyanide degradation. As it is shown in Fig. 9a, complete destruction of cyanide was accomplished only after 30 min, and the highest *K*_obs_ is observed by this process. The higher removal efficiency of MgO/O_3_/PS could be attributed to the above-mentioned mechanisms which are simultaneously occurring in the solution to generate sulfate and hydroxyl radicals.

Comparison of the *K*_obs_ of the process in the presence of EtOH shows much more reduction than that of TBA. Therefore, formation of both SO_4_˙^−^ and HO˙ are proved. Table 3 is also showing that SO_4_˙^−^ contribution for cyanide oxidation is 37.5% and HO˙ is 62.5% in the MgO/O_3_/PS process. Accordingly, it can be concluded that MgO/O_3_/PS is a promising process for cyanide degradation.

The amount of consumed ozone was also investigated in MgO/O_3_/PS process, and about 25.5% ozone was measured to be used in this process which is the highest amount of consumed ozone in the present study. The higher consumption of ozone in the MgO/O_3_/PS process is due to the presence of various ozone quenchers including the alkaline cyanide solution, MgO catalyst, and persulfate.

No report was found in the literature of a catalytic ozonation of cyanide using MgO to compare the results. As well as, the combination method of MgO/O_3_/PS has not yet been tested on degradation of cyanide. However, there are some other advanced oxidation processes which have been reported in literature for cyanide oxidation, and the results of present study is comparable with them. Guo *et al.*^60^ investigated photoelectrocatalytic degradation of cyanide in the presence of PS. TiO_2_ nanorods and CuFe_2_O_4_ modified graphite felt were used as anode and cathode, respectively. They have reported that 0.9 mM of cyanide was almost completely degraded after 120 min of reaction time. The *K*_obs_ value under the optimum condition of the process was calculated to be 0.0314 min^−1^. Budaev *et al.*^61^ investigated the performance sulfate based AOPs for thiocyanate degradation. They have investigated various oxidation process such as PS + *T* (50 °C), PS/Fe^2+^ and PS/Fe^3+^ for oxidation of 1.72 mM thiocyanate. The results show that heating the solution to 50 °C and addition PS alone was not effective for thiocyanate degradation and only 7% removal efficiency has been reached. While PS/Fe^2+^ and PS/Fe^3+^ were able to completely destruct thiocyanate with reaction rate constants of 0.058 and 0.096 min^−1^, respectively. Finally, it can be deduced that MgO/O_3_/PS process, in comparison with other proposed techniques, is an effective technique for simultaneous generation of sulfate and hydroxyl radical to degrade cyanide.

### 3.7. The effect of different water matrices and real wastewater treatment

**Fig. 11:**
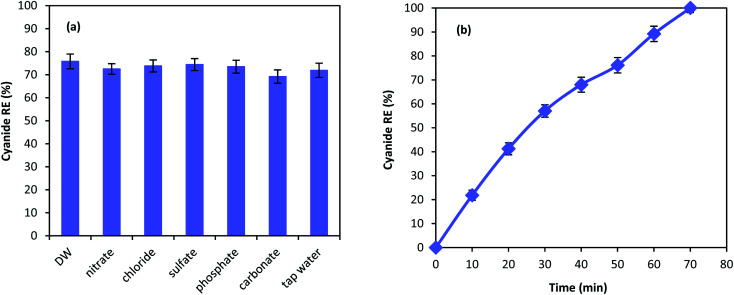
Cyanide degradation in the presence of anions (a) and real industrial wastewater (b).

Chemical degradation of contaminants are highly affected by various compounds present in the solution.^62^ Therefore, the effect of different anions on the performance of MgO/O_3_/PS process was investigated. 100 mg L^−1^ of nitrate, chloride, sulfate, phosphate, and carbonate along with tap water were used in the MgO/O_3_/PS process. Fig. 11a is illustrating the effect anions on the process performance. As shown in the figure, inorganic compounds are not capable of deteriorating the process performance and only a slight reduction has been observed in cyanide removal. Furthermore, cyanide oxidation in tap water compared to that of distilled water is not effected by the water ingredients, proving the fact that MgO/O_3_/PS process can be reliable in different water matrices. Even though anions are not considered as limitation for this process, cyanide removal efficiency has slightly been reduced in presence of carbonate. Anions such as carbonate is dependent to the Lewis acids and they can occupy the active sites of the catalysts surface. Therefore, reduction in ozone decomposition on the catalyst surface takes place.^63^ However, the reduction in the presence of inorganic anions is negligible. Accordingly, it can be inferred that generated radicals are released into the bulk solution to react with the cyanide molecules, and the oxidation reaction mainly occur in the bulk solution rather than catalyst surface.

Fig. 11b is showing the performance of the MgO/O_3_/PS in the real industrial wastewater. Cyanide oxidation in the real wastewater is considerably affected by the real wastewater matrix, and a substantial reduction in cyanide removal efficiency has been observed. However, complete cyanide oxidation is also reached in the complicated matrix of industrial wastewater, after reaction time of 70 min. The reduction in the process performance is due to the presence of other organic radical quenchers in the wastewater that lead to the reduction in removal efficiency of our target pollutant.

### 3.8. Cyanide degradation by-products

In order to see the fate of cyanide molecules in the investigated process, the possible cyanide degradation by-products were measured. Degradation by-products were measured at two different times of 30 min (complete cyanide degradation was reached) and 60 min to see the degradation of carbon and nitrogen-based compounds. Based on previous studies cyanide degradation may lead to the generation of cyanate, nitrate, nitrite, ammonium, nitrogen gas and carbon dioxide. In addition, to cyanide degradation by products, sulfate and residual persulfate were also measured in the process effluent. Table 4 is giving the measured by-products of cyanide degradation at 30 and 60 min of reaction time. As it is shown, after 30 min, complete degradation of cyanide has been reached, and formation of degradation by-products are observed. Cyanate and ammonium are the main primarily generated by-products. Accordingly, two distinct degradation pathways are proposed for cyanide degradation (eqn (10)–(12)): (1) degradation of cyanide through cyanate oxidation, (2) degradation of cyanide through ammonium oxidation.

10 CN^−^ + oxidizing agents → OCN^−^ + NH_4_^+^

11 OCN^−^ + oxidizing agents → HCO_3_^−^/CO_3_^2−^ + CO_2_

12 NH_4_^+^ + oxidizing agents → NO_2_^−^ + NO_3_^−^ + N_2_

**Table 4:** Cyanide degradation by-products in MgO/O_3_/PS process (values are in mg L^−1^)

Chemical compound	*T* = 0 min	*T* = 10 min	*T* = 20 min
CN	100	0	0
OCN^−^	0	61.3	0
NH_4_^+^	0	15.4	0
NO_3_^−^	0	16.1	18.7
NO_2_^−^	0	19.3	1.3
HCO_3_^−^/CO_3_^2−^	0	443	510
S_2_O_8_^2−^	200	43.8	15.2
SO_4_^2−^	0	150.9	180.4

As it is shown in the table, cyanate is formed after 30 min, while it is completely oxidized after 60 min, and carbonate concentration has increased in the process. Therefore, carbon content of cyanide primarily transfers to cyanate and further exposure of the solution with oxidizing agents transfers the carbon to carbonate. Based on previous studies, cyanide oxidation may also lead to the generation of carbon dioxide.^64^

In the second degradation pathway, ammonium concentration has increased after cyanide degradation. Further increase of the reaction time leads to formation of other nitrogenous compounds such as nitrite and nitrate, and complete elimination of ammonium is reached. On the other hand, nitrite was primarily produced which has changed to nitrate. Finally, nitrate was the dominant nitrogenous compound in the solution and only a negligible amount of nitrite is remaining in the solution. Comparing the results of cyanide oxidation with similar studies shows a similar trend in carbonaceous and nitrogenous compounds of the effluent.^9,15,64–66^ Kim *et al.*^64^ investigated cyanide degradation in UV/TiO_2_ process and tracked the final by-products. They also reported generation of cyanate as the intermediate product and nitrate, nitrite and carbonate were the final generated by-products. They also measured carbon dioxide and nitrogen gas in the off-gas of the process which are generated as a result of cyanide oxidation. Moussavi *et al.*^15^ also proved cyanate and ammonium generation as a result of cyanide oxidation, but finally, they all were converted to carbonate and nitrate in the effluent. There is only one controversy in the literature in various types of oxidation process which is in case of ammonium generation. Yeddou *et al.*^66^ reported the formation of ammonium ions as the intermediate products which is stated to be due to the presence of redundant amount of H_2_O_2_ as an auxiliary oxidant in the solution. On the other hand, Kim *et al.*^64^ only have detected nitrite, nitrate and nitrogen gas as the by-products of cyanide oxidation. However, in our study, ammonium was detected as the intermediate product.

Persulfate was the chemical additive in the present study to accelerate process performance. The fate of persulfate was also tracked in the present study. It was found that persulfate is consumed in this process and finally it converts to sulfate ion in the effluent. Accordingly, it can be deduced that MgO/O_3_/PS process is a reliable method for conversion of toxic cyanide to much less noxious compounds.

### 3.9. Catalyst reusability

**Fig. 12:**
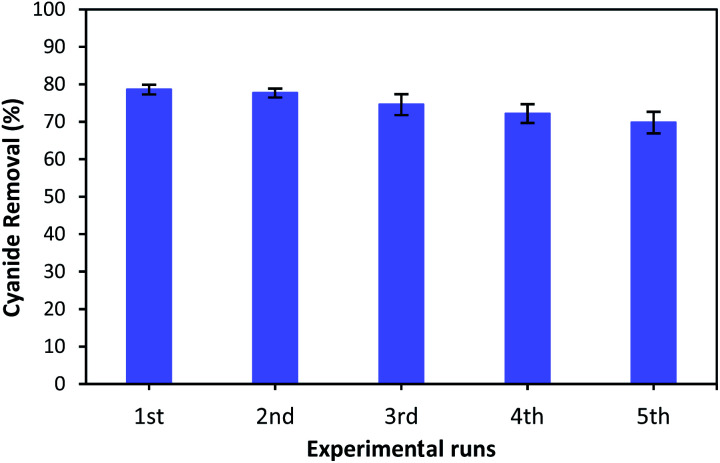
Reusability of MgO catalyst for the removal of cyanide.

As the reusability of the catalyst is very important factor from the practical, environmental and economic viewpoint,^23,67^ the catalytic reusability of MgO was studied in a consecutive reusability test. To recover the MgO catalyst, the solution containing catalyst was centrifuged at 5000 rpm for 10 minutes after each use. The removed catalyst was washed several times with distilled water, dried at 105 °C for 24 hours, and reused for the next reaction. Fig. 12 illustrates the cyanide removal efficiency when using the recycled and regenerated MgO catalyst in five batch runs. It can be seen that the cyanide removal potential was 78.6% when regenerated catalyst was used for the first time. The removal rate decreased slightly after each cycle. However, the cyanide removal capacity was still 69.8% after 5 cycles indicating that MgO shows a relatively stable catalytic performance for the COP experiment. A little decrease in removal capacity can be ascribed to the occupation of surface active sites with some impurities, and the mass loss of MgO during the recovery process in each cycle. However, the catalytic activity of regenerated MgO remained as high as 69.8% after 5 runs. Therefore, these results reveal that synthesized catalyst can be considered as a relatively stable catalyst in the catalytic ozonation of cyanide.

## 4. Conclusion

Present study investigated catalyzed ozonation process with MgO nanoparticles and the process performance was accelerated by addition of persulfate as sulfate based radical precursors. The operating parameters in the MgO/O_3_/PS process were completely investigated and a comprehensive investigation was conducted to find the cyanide degradation mechanism. The following conclusions can be mentioned for this study:

• The synthesized catalyst with nanocrystalline structure had specific surface area of 198.3 m^2^ g^−1^ and average particle size of 7.42 nm.

• Catalyzing ozonation process had almost 30% synergy for cyanide degradation in MgO/O_3_.

• The highest cyanide removal rate was observed in the presence of 400 mg h^−1^ injected ozonation, 200 mg L^−1^ persulfate and 600 mg L^−1^ MgO catalyst. Almost 25.5% of the injected ozone was consumed in the MgO/O_3_/PS.

• Five distinct mechanisms (SOP, MgO/O_3_, PS/O_3_, MgO/PS, and MgO/O_3_/PS) were proposed and proved for cyanide oxidation.

• Scavenging experiments proved generation of both sulfate and hydroxyl radicals in the MgO/O_3_/PS process.

• The MgO/O_3_/PS process was able to oxidize 173 mg L^−1^ of cyanide after 70 min of reaction time in real industrial wastewater.

• The final generated by-products of cyanide oxidation were tracked and it was found that toxic cyanide has completely destructed to much less toxic compounds such as nitrate and carbonate.

Finally, according to the findings of the present study and proposed mechanisms and degradation pathways, it can be concluded that catalyzed ozonation process with MgO nanoparticles and persulfate is a reliable advanced oxidation process for removal of cyanide from industrial wastewater.

## Author contributions

Ali Behnami: conducting experiments, data curation and writing the manuscript. Jean-Philippe Croué: writing and revising the manuscript. Ehsan Aghayani: conceptualization and conducting experiments. Mojtaba Pourakbar: conceptualization, methodology, acquisition of the financial support for the project, writing the manuscript.

## Conflicts of interest

There are no conflicts to declare.

## Supplementary Material

## References

[cit1] Matino I., Colla V., Branca T. A. (2018). Front. Chem. Sci. Eng..

[cit2] Li Q., Lu H., Yin Y., Qin Y., Tang A., Liu H., Liu Y. (2019). J. Hazard. Mater..

[cit3] Noroozi R., Al-Musawi T. J., Kazemian H., Kalhori E. M., Zarrabi M. (2018). J. Water Process. Eng..

[cit4] Moussavi G., Khosravi R. (2010). J. Hazard. Mater..

[cit5] Kaewkannetra P., Imai T., Garcia-Garcia F. J., Chiu T. Y. (2009). J. Hazard. Mater..

[cit6] Maulana I., Takahashi F. (2018). J. Water Process. Eng..

[cit7] Mondal M., Mukherjee R., Sinha A., Sarkar S., De S. (2019). J. Water Process. Eng..

[cit8] Dash R. R., Gaur A., Balomajumder C. (2009). J. Hazard. Mater..

[cit9] Moussavi G., Pourakbar M., Aghayani E., Mahdavianpour M., Shekoohyian S. (2016). Chem. Eng. J..

[cit10] Moussavi G., Pourakbar M., Shekoohiyan S., Satari M. (2018). Chem. Eng. J..

[cit11] Li R., Kong J., Liu H., Chen P., Liu G., Li F., Lv W. (2017). RSC Adv..

[cit12] Li H., Yang Y.-L., Li X., Fan X.-Y., Wang N. (2021). J. Environ. Chem. Eng..

[cit13] Mudliar R., Umare S. S., Ramteke D. S., Wate S. R. (2009). J. Hazard. Mater..

[cit14] Mert B. K., Sivrioğlu Ö., Yonar T., Özçiftçi S. (2014). Ozone: Sci. Eng..

[cit15] Moussavi G., Pourakbar M., Aghayani E., Mahdavianpour M. (2018). Chem. Eng. J..

[cit16] Budaev S. L., Batoeva A. A., Tsybikova B. A. (2014). J. Environ. Chem. Eng..

[cit17] Kim T.-K., Kim T., Jo A., Park S., Choi K., Zoh K.-D. (2018). Chemosphere.

[cit18] Hernández-Alonso M. D., Coronado J. M., Soria J., Conesa J. C., Loddo V., Addamo M., Augugliaro V. (2007). Res. Chem. Intermed..

[cit19] Wu K., Zhang F., Wu H., Wei C. (2018). Environ. Sci. Pollut. Res..

[cit20] Liu Z.-Q., Tu J., Wang Q., Cui Y.-H., Zhang L., Wu X., Zhang B., Ma J. (2018). Sep. Purif. Technol..

[cit21] Giagnorio M., Amelio A., Grüttner H., Tiraferri A. (2017). J. Cleaner Prod..

[cit22] Moussavi G., Khosravi R., Omran N. R. (2012). Appl. Catal.,
A.

[cit23] Mashayekh-Salehi A., Moussavi G., Yaghmaeian K. (2017). Chem. Eng. J..

[cit24] Luo H., Wang Y., Wen X., Cheng S., Li J., Lin Q. (2021). Sci. Total Environ..

[cit25] Zhou J., Xia Y., Gong Y., Li W., Li Z. (2020). Sci. Total Environ..

[cit26] Alinejad A., Akbari H., Ghaderpoori M., Jeihooni A. K., Adibzadeh A. (2019). RSC Adv..

[cit27] He K., Dong Y. M., Li Z., Yin L., Zhang A. M., Zheng Y. C. (2008). J. Hazard. Mater..

[cit28] Chen J., Tian S., Lu J., Xiong Y. (2015). Appl. Catal., A.

[cit29] Moussavi G., Mahmoudi M. (2009). Chem. Eng. J..

[cit30] Fouda A., Hassan S. E.-D., Saied E., Hamza M. F. (2021). J. Environ. Chem. Eng..

[cit31] El-Shamy A. G. (2020). Polymer.

[cit32] APHA/AWWA/WEF , Standard Methods for the Examination of Water and Wastewater, American Public Health Association, Washington, DC, USA, 20th edn, 2005

[cit33] Güpta Y. K. (1961). Fresenius' Z. Anal. Chem..

[cit34] Holzwarth U., Gibson N. (2011). Nat. Nanotechnol..

[cit35] Balakrishnan G., Velavan R., Mujasam Batoo K., Raslan E. H. (2020). Results Phys..

[cit36] Ercan I., Kaygili O., Ates T., Gunduz B., Bulut N., Koytepe S., Ozcan I. (2018). Ceram. Int..

[cit37] Karthik K., Dhanuskodi S., Gobinath C., Prabukumar S., Sivaramakrishnan S. (2019). J. Photochem. Photobiol., B.

[cit38] Mohandes F., Davar F., Salavati-Niasari M. (2010). J. Phys. Chem. Solids.

[cit39] Selvam N. C. S., Kumar R. T., Kennedy L. J., Vijaya J. J. (2011). J. Alloys Compd..

[cit40] Fakhri A., Adami S. (2014). J. Taiwan Inst. Chem. Eng..

[cit41] Chowdhury I. H., Chowdhury A. H., Bose P., Mandal S., Naskar M. K. (2016). RSC Adv..

[cit42] Cai Y., Li C., Wu D., Wang W., Tan F., Wang X., Wong P. K., Qiao X. (2017). Chem. Eng. J..

[cit43] Yu X.-Y., Luo T., Jia Y., Zhang Y.-X., Liu J.-H., Huang X.-J. (2011). J. Phys. Chem. C.

[cit44] Zhu D., Chen Y., Yang H., Wang S., Wang X., Zhang S., Chen H. (2020). Chemosphere.

[cit45] Shu H.-Y., Chang M.-C., Huang S.-W. (2015). Desalin. Water Treat..

[cit46] Castilla-Acevedo S. F., Betancourt-Buitrago L. A., Dionysiou D. D., Machuca-Martínez F. (2020). J. Hazard. Mater..

[cit47] Das P. P., Anweshan A., Mondal P., Sinha A., Biswas P., Sarkar S., Purkait M. K. (2021). Chemosphere.

[cit48] Kasprzyk-Hordern B., Ziółek M., Nawrocki J. (2003). Appl. Catal., B.

[cit49] Weizhou J., Youzhi L., Wenli L., Jing L., Fan S., Chaoran W. (2013). China Pet. Process. Petrochem. Technol..

[cit50] Khoshnamvand N., Jafari A., Kamarehie B., Mohammadi A., Faraji M. (2019). Environ. Processes.

[cit51] Pourakbar M., Moussavi G., Shekoohiyan S. (2016). Ecotoxicol. Environ. Saf..

[cit52] Aramendía M. a. A., Borau V., Jiménez C., Marinas J. M., Ruiz J. R., Urbano F. J. (2003). Appl. Catal., A.

[cit53] Sánchez-Polo M., von Gunten U., Rivera-Utrilla J. (2005). Water Res..

[cit54] Zhang T., Li C., Ma J., Tian H., Qiang Z. (2008). Appl. Catal., B.

[cit55] Qiao J., Luo S., Yang P., Jiao W., Liu Y. (2019). J. Taiwan Inst. Chem. Eng..

[cit56] Abu Amr S. S., Aziz H. A., Adlan M. N., Bashir M. J. K. (2013). Chem. Eng. J..

[cit57] Oh W.-D., Dong Z., Lim T.-T. (2016). Appl. Catal., B.

[cit58] Zhang Y., Zhang Q., Hong J. (2017). Appl. Surf. Sci..

[cit59] Guan Y.-H., Ma J., Ren Y.-M., Liu Y.-L., Xiao J.-Y., Lin L.-q., Zhang C. (2013). Water Res..

[cit60] Guo T., Dang C., Tian S., Wang Y., Cao D., Gong Y., Zhao S., Mao R., Yang B., Zhao X. (2018). Chem. Eng. J..

[cit61] Budaev S. L., Batoeva A. A., Tsybikova B. A. (2015). Miner. Eng..

[cit62] Moussavi G., Rezaei M., Pourakbar M. (2018). Chem. Eng. J..

[cit63] Rashidashmagh F., Doekhi-Bennani Y., Tizghadam-Ghazani M., van der Hoek J. P., Mashayekh-Salehi A., Heijman B. S. G. J., Yaghmaeian K. (2021). J. Hazard. Mater..

[cit64] Kim S. H., Lee S. W., Lee G. M., Lee B.-T., Yun S.-T., Kim S.-O. (2016). Chemosphere.

[cit65] Malhotra S., Pandit M., Kapoor J., Tyagi D. (2005). J. Chem. Technol. Biotechnol..

[cit66] Yeddou A. R., Nadjemi B., Halet F., Ould-Dris A., Capart R. (2010). Miner. Eng..

[cit67] Hanafi M. F., Sapawe N. (2020). Mater. Today: Proc..

